# Pulmonary neuroendocrine cells: crucial players in respiratory function and airway-nerve communication

**DOI:** 10.3389/fnins.2024.1438188

**Published:** 2024-08-08

**Authors:** Abhimanyu Thakur, Shuya Mei, Noel Zhang, Kui Zhang, Boghos Taslakjian, Jiacee Lian, Shuang Wu, Bohao Chen, Julian Solway, Huanhuan Joyce Chen

**Affiliations:** ^1^Pritzker School of Molecular Engineering, The University of Chicago, Chicago, IL, United States; ^2^Ben May Department for Cancer Research, The University of Chicago, Chicago, IL, United States; ^3^Canyon Crest Academy, San Diego, CA, United States; ^4^School of Health Sciences, Ngee Ann Polytechnic, Singapore, Singapore; ^5^Department of Medicine, Section of Pulmonary and Critical Care Medicine, The University of Chicago, Chicago, IL, United States

**Keywords:** pulmonary neuroendocrine cells, lung, brain, stem cells, HPSC, IPSC

## Abstract

Pulmonary neuroendocrine cells (PNECs) are unique airway epithelial cells that blend neuronal and endocrine functions, acting as key sensors in the lung. They respond to environmental stimuli like allergens by releasing neuropeptides and neurotransmitters. PNECs stand out as the only lung epithelial cells innervated by neurons, suggesting a significant role in airway-nerve communication via direct neural pathways and hormone release. Pathological conditions such as asthma are linked to increased PNECs counts and elevated calcitonin gene-related peptide (CGRP) production, which may affect neuroprotection and brain function. CGRP is also associated with neurodegenerative diseases, including Parkinson’s and Alzheimer’s, potentially due to its influence on inflammation and cholinergic activity. Despite their low numbers, PNECs are crucial for a wide range of functions, highlighting the importance of further research. Advances in technology for producing and culturing human PNECs enable the exploration of new mechanisms and cell-specific responses to targeted therapies for PNEC-focused treatments.

## Introduction

Breathing exposes our lungs to numerous external elements, such as ambient aerosols, pathogens, allergens, and pollutants (Wang et al., 2021). Therefore, the lungs have evolved various defense mechanisms, including immune signaling and neuronal circuit activation (Schiller et al., 2021; Udit et al., 2022). In 1949, Fröhlich’s discovery of pulmonary neuroendocrine cells (PNECs), termed ‘helle zellen’ or ‘bright cells’ in German, revealed the cell type responsible for detecting harmful agents (Frohlich, 1949). Following this, in 1954, Feyrter introduced the concept of the diffuse neuroendocrine system, further expanding the understanding of these critical cellular functions (Feyrter, 1954). Lauweryns and colleagues discovered innervated neuroepithelial bodies (NEBs) in the lungs of 15 human infants, a finding later corroborated in rabbits in 1970s (Lauweryns and Peuskens, 1972; Lauweryns et al., 1973). As part of the neuroendocrine system, PNECs are rare but evolutionarily conserved epithelial cells in air-breathing vertebrates, comprising less than 0.5% of total airway epithelial cells (Boers et al., 1996). PNECs are different from other neuroendocrine cells in various aspects as summarized in Table 1. PNECs are found throughout the airways, either as solitary cells or in clusters known as NEBs. They are uniquely innervated by both sensory (afferent) and motor (efferent) nerve fibers, primarily of vagal origin. This innervation increases during gestation and involves factors like neurotrophin 4. The extensive innervation sets PNECs apart from other lung progenitor cells, enabling their function as airway chemoreceptors.(Linnoila, 2006).

**TABLE 1:** Major differences between PNECs and other endocrine cells.

Feature	PNECs	Other Endocrine Cells
Location	Airways of the lungs (scattered and in clusters called neuroepithelial bodies)	Diverse, including glands (thyroid, pancreas), digestive tract, kidneys, etc.
Primary Function	Sensory and paracrine signaling: Monitor airway status and regulate breathing	Hormone production: Secrete hormones into the bloodstream to regulate various bodily functions.
Signaling Molecules	Neuropeptides and neurotransmitters: Serotonin, calcitonin, CGRP, GABA, etc.	Hormones: Insulin, glucagon, adrenaline, thyroxine, etc.
Release Mechanism	Direct release into airway lumen or paracrine secretion to nearby cells: Local effects on airway muscles and nerves	Release into bloodstream for effects on distant organs: Long-range effects on various body systems
Development	Derived from the same progenitor cells as other lung epithelial cells	Derived from specialized embryonic tissues
Regeneration	Limited regenerative capacity	Varying regenerative capacity depending on the specific cell type
Clinical Significance	Potential role in lung development, injury, and tumorigenesis (e.g., small-cell lung cancer)	Involved in various endocrine disorders and some cancers

PNECs are seen as pivotal chemo-sensitive cells in detecting various chemical and physical stimuli in the airways (Cutz et al., 2013; Poirier et al., 2020). Their roles extend to regulating airway tone, mucociliary clearance, and contributing to bronchial hyperresponsiveness and asthma development (Xu et al., 2020; Hewitt and Lloyd, 2021; Mou et al., 2021; Kuo et al., 2022). PNECs can secrete hormones and signaling molecules as a component of the endocrine system, owing to their derivation from neuroendocrine progenitor cells located in the airway epithelium (Garg et al., 2019). These hormones have multiple downstream effects, including inducing epinephrine and cortisol and managing mucus production and inflammatory responses (Li et al., 2023). Recent research has associated PNECs with lung cancer development, specifically small cell lung cancer (SCLC), the most aggressive type (Schuller et al., 2003). PNECs are considered as the cell origin for SCLC, and this association is believed to arise from alterations in the genes or signaling pathways commonly found in SCLC that regulate PNECs growth and differentiation, such as RB, TP53 and NOTCH (Chen et al., 2019).

Recent studies have suggested PNECs also interact with the brain, affecting physiological functions like breath and heart rate (Kuo et al., 2022). This communication occurs through the vagus nerve, a long nerve connecting the brain to the lung and abdomen and influencing various bodily functions. When stimulated, PNECs secrete various neuropeptides. These neuropeptides then traverse through the vagus nerve to reach the brain, where they modulate the brainstem centers responsible for regulating respiration (Branchfield et al., 2016). Furthermore, PNECs can communicate with the brain through the bloodstream, with hormones like CGRP enhancing brain blood flow and cognitive function (Gu et al., 2014). This interaction is a vital part of the body’s homeostatic control system, maintaining internal stability amidst external environmental changes. Hypoxia can prompt PNECs to signal the brain, increasing the breathing rate to ensure adequate oxygen supply (Branchfield et al., 2016; Li et al., 2023). Despite their rarity in the lungs, PNECs play crucial roles in diverse physiological and pathological conditions, marking them as a significant research area. Their involvement in various lung health and disease aspects, serving as a vital link between the lung and brain, will be explored further in subsequent sections.

## Contribution of PNECs to the lung physiological function

**FIGURE 1:**
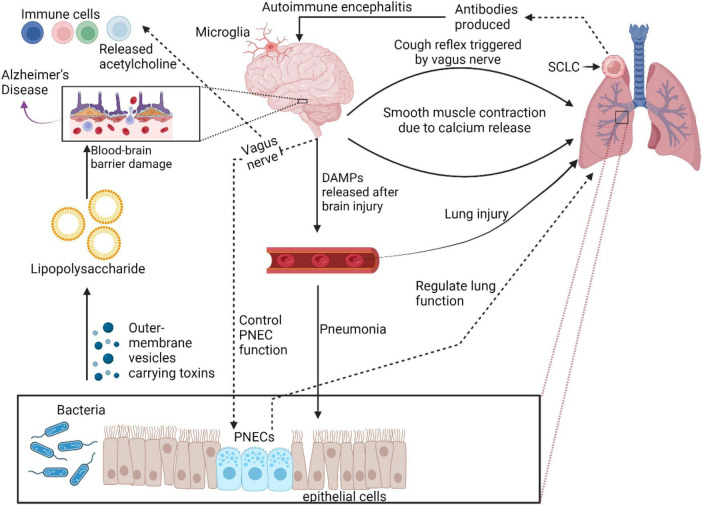
Role of PNECs in physiological and pathological conditions in lung. PNECs play crucial role in lung physiology and pathology. They function as sensory components, sensing oxygen and chemical stimuli, and are involved in oxygen sensing and mechano-transduction. PNECs are implicated in various lung diseases, regulating immune responses and tissue remodeling. Their diverse signaling output and release of neuropeptides make them critical sensors in the lung, influencing respiratory function and disease pathogenesis.

PNECs are predominantly located as NEBs or single cells in the airway epithelium of the lungs (Song et al., 2012; Kuo and Krasnow, 2015), as shown in Figure 1. PNECs display a remarkable capacity for cellular fate transformation in response to lung injury, underscoring their plasticity. During lung development, PNECs and alveolar cells originate from different progenitor lineages (Desai et al., 2014). In the context of injury, PNECs contribute to the formation of club and ciliated cells (Song et al., 2012; Lee et al., 2017; Yao et al., 2018). This suggests that PNECs represent a type of progenitor cell, alongside other known progenitors like basal cells, club cells, and type II cells, all capable of differentiating into various cell types under specific conditions (Song et al., 2012; Branchfield et al., 2016; Lee et al., 2017). The nature of a lung insult dictates the unique tissue response, with PNECs being potentially induced to differentiate into additional cell types in reaction to diverse forms of injury. Notably, PNECs are irreplaceable once lost during homeostasis or certain lung injuries, although some forms of lung injury may trigger their regeneration (Song et al., 2012; Yao et al., 2018; Shue et al., 2022).he mechanisms driving PNECs proliferation and fate alteration in these varied scenarios are yet to be fully elucidated.

PNECs play a crucial role in various lung physiological functions such as respiratory control, immune defense, vascular regulation, and mechanical stress detection (Chen et al., 2019). For example, PNECs regulate blood flow by releasing CGRP, a powerful vasodilator that relaxes blood vessel walls, enhancing lung circulation. Via direct communication with the central neuro system, PNECs help to adjust breathing rates, ensuring adequate oxygen supply, particularly under challenging conditions. PNECs also contribute to the immune modulation of the lungs by secreting hormones and peptides such as CGRP, serotonin, and gastrin-releasing peptide, controlling inflammation and enhancing the proliferation of protective immune cells (Gu et al., 2014). Additionally, the chemosensitivity of PNECs allows them to detect and respond to airborne pollutants, allergens, as well as airway damage (Kuo et al., 2022), triggering appropriate protective actions (Gu et al., 2014).

## Role of PNECs in different lung diseases

### Small cell lung cancer

PNECs are associated with the development and advancement of SCLC, a highly aggressive type of lung cancer (Sutherland et al., 2011; Semenova et al., 2015). Targeting PNECs with CGRP-Cre and using CGRP-CreER PNEC lineage-tracking mice further confirmed SCLC’s origin from PNECs (Sutherland et al., 2022). Furthermore, mutations in Notch pathway genes, identified in a substantial portion of SCLC patients, highlight the significant role of this pathway in the development of SCLC. Consequently, the Notch pathway and its modulation have become potential therapeutic targets for SCLC (Augert et al., 2019).

NEBs house a small subset of PNECs, which are proposed to function as reserve stem cells (NE^stem^)(Ouadah et al., 2019). Deletion of Rb and p53 in mouse PNECs has been shown to lead to the formation of similar tumors, suggesting a potential link to SCLC. However, in this model, transformation occurs in only a rare subset of PNECs cells. It has been proposed that NE^stem^, a specific subgroup of PNECs, could be the primary originators of SCLC initiation is thought to stem from the immediate and sustained activation of NE^stem^ cell renewal after the loss of Rb and p53.This loss might render NE^stem^ cells unresponsive to signals that typically trigger their dispersal or revert them to a non-cancerous state, potentially explaining the propensity of SCLC for early metastatic spread. Conversely, an increased responsiveness to these deprogramming signals could result in less malignant outcomes, potentially acting as a form of tumour suppression. This theory might shed light on the frequent occurrence of Notch receptor loss-of-function mutations in human SCLCs, which prevent deprogramming and thereby maintain the NE^stem^ identity of the tumors (Ouadah et al., 2019).

### Asthma

PNECs have been increasingly implicated in the pathogenesis of asthma (Sui et al., 2018). In exercise-induced asthma, PNEC might conceivably sense the osmolar and thermal changes in airway and cause reflex parasympathetic stimulation, leading to bronchoconstriction via the vagus nerve. PNECs are in close proximity to the subjacent airway smooth muscle. Activation of PNECs in asthmatic patients leads to the release of molecules that contribute to inflammation and airway hyperreactivity. They release CGRP, a bronchoconstrictor, and γ-aminobutyric acid (GABA), which induces goblet cell hyperplasia. PNECs also interact with immune cells like ILC2s, stimulating them to produce inflammatory cytokines (IL-5, IL-13), exacerbating asthmatic symptoms. Another research group suggested that PNECs are highly likely to cause mucus hypersecretion via stimulating GABAergic system and eventually delay the recovery from respiratory diseases including asthma (Piao et al., 2021).These emerging evidences suggest that targeting PNECs could be a promising therapeutic strategy for treating asthma (Barrios et al., 2019). Further research is required to comprehensively understand their role in this condition.

### Viral infection

Understanding the interaction between PNECs and viruses is essential in addressing respiratory diseases. Notably, the COVID-19 pandemic, caused by the SARS-CoV-2 virus, resulted in significant global health challenges, with over 770 million cases and 7 million deaths by the end of 2023, according to the World Health Organization (WHO) COVID-19 dashboard. Despite the WHO declared the end of COVID-19’s emergency phase in May 2023, research into the disease continues. Interestingly, PNECs, despite its lack of the ACE2 receptors typically used by the virus for cell entry (Peng et al., 2022), are suspected to influence the severe immune responses observed in COVID-19 (Noguchi et al., 2020). PNECs respond to hypoxia and possess chemosensory receptors, potentially linking PNECs to early COVID-19 symptoms and long-term effects such as anosmia and dysgeusia in long-COVID patients (Caretta and Mucignat-Caretta, 2021; Shivaraju et al., 2021).

In the context of influenza, another significant public health concern, PNECs play a proactive role during infections. PNECs secrete gastrin-releasing peptide (GRP), which can provoke pro-inflammatory responses that contribute to lung damage (Shirey et al., 2019). Targeting GRP or its receptor might, therefore, reduce such damage. Additionally, the release of GABA from PNECs during influenza infection further supports their role in inflammatory responses (Shirey et al., 2023). A deeper understanding of PNECs in viral infections could lead to innovative treatments that target PNECs directly or modulate their secretions to mitigate disease symptoms and progression.

### Bacterial and fungal infection

The interactions between PNECs and bacterial or fungal infections in the lungs are an emerging area of research. While specific interactions are not extensively documented, existing knowledge about PNECs suggests possible roles. As we mentioned before, PNECs often hyperactivate in lung diseases such as SCLC (Chen et al., 2019; Poirier et al., 2020; Sutherland et al., 2022), asthma (Sui et al., 2018), and viral infections (Shirey et al., 2019; Caretta and Mucignat-Caretta, 2021, 2022; Shirey et al., 2023). PNECs play a role in sensing environmental changes like hypoxia and chemo-stress, and in regulating immune responses, including Type 2 immune responses and allergic reactions. While PNECs’ direct and indirect roles in bacterial and fungal infections require more research, it’s likely they contribute to the lung’s response to these pathogens (Bordon, 2016; Branchfield et al., 2016; Kobayashi and Tata, 2018; Sui et al., 2018; Hewitt and Lloyd, 2021). Further studies are essential to understand these interactions and their impact on lung health and disease management.

### Congenital lung diseases

Recent research highlights the significant role of PNECs in various congenital lung diseases such as neuroendocrine hyperplasia of infancy (NEHI), bronchopulmonary dysplasia (BPD), and congenital diaphragmatic hernia (CDH) (Cutz et al., 2007; Cutz, 2015; Noguchi et al., 2020). These studies reveal that PNECs are critical in lung development and the inflammatory processes that regulate cell growth and death, essential for proper lung structure formation.

NEHI is a rare pediatric lung disorder mainly affecting infants, characterized by symptoms like rapid breathing and chronic low oxygen levels, detectable through specific CT scan patterns (Spielberg et al., 2021). Although the cause of NEHI remains unclear, diagnoses typically involve lung biopsies that reveal an increase in specific PNECs, indicating hyperplasia without other significant lung abnormalities or inflammation (Cutz et al., 2007; Young et al., 2011). Despite extensive study, the role of these PNECs in NEHI remains poorly understood. However, genetic research, including studies on familial groups and mouse models, suggests that the NKX2.1 gene may be crucial in understanding the impact of PNECs in NEHI (Li et al., 2013; Young et al., 2013; Nevel et al., 2016).

In BPD, particularly common among premature infants subjected to prolonged mechanical ventilation and high oxygen levels (Johnson et al., 1982; Sunday et al., 2004), both traditional forms characterized by extensive epithelial injury and fibrosis, and newer forms known as “post-surfactant BPD,” show significant PNEC hyperplasia (Northway et al., 1967; Gillan and Cutz, 1993; Husain et al., 1998). The hyperplasia of PNECs, driven by persistent hypoxia and inflammatory signals, promotes increased cellular proliferation affecting various pathological aspects of BPD, such as inflammation and disrupted lung structure (Cutz, 2015; Martin et al., 2015). Research using animal models has shown that targeting the bombesin-like peptides (BLP) secreted by these PNECs can alleviate BPD symptoms, highlighting the significant role of PNEC hyperplasia in the disease’s progression and its potential as a therapeutic target (Subramaniam et al., 2003, 2007; Ashour et al., 2006).

In CDH, impaired lung development due to diaphragmatic defects also involves PNEC dysfunction. Increased BLP production by PNECs, which act similarly to growth factors, has been observed in CDH-affected lungs, reflecting their importance in lung maturation and the potential impacts of their altered function on lung development (Ijsselstijn et al., 1997, 1998; Asabe et al., 1999). This evidence underscores the crucial role of PNECs in congenital lung diseases and points to their potential as targets for therapeutic interventions.

### Other lung pathological conditions

PNECs are increasingly recognized for their role in chronic obstructive pulmonary disease (COPD) and bronchiectasis. In COPD patients, PNECs are more numerous and may contribute to airway inflammation and remodeling by releasing mucus-producing and inflammatory factors. Similarly, in bronchiectasis, the elevated number of PNECs could be linked to airway damage and inflammation. While PNECs are also implicated in other lung diseases like pneumonia and acute respiratory distress syndrome, further research is necessary to fully understand their functions in these conditions. Current evidence, however, underscores their significant role in the development and progression of various lung diseases (Randell, 2006; Bordon, 2016; Noguchi et al., 2020)

## Role of PNECs in airway and nervous system communications

PNECs connect with the brain through direct neural pathways and by releasing neurotransmitters and hormones (Linnoila, 2006; Kuo et al., 2022; Li et al., 2023). NEBs interact closely with sensory nerve terminals. Myelinated afferent nerves branch into NEBs, while unmyelinated sensory nerves from the dorsal root ganglia also innervate these structures. These axons transmit sensory information to the brainstem. NEBs detect CO2, air pressure, O2, H + ions, and nicotine, triggering various physiological responses (Noguchi et al., 2020). PNECs have neural connections to the brainstem and spinal cord, enabling them to influence vital functions like breathing and heart rate. PNECs release neurotransmitters such as serotonin, norepinephrine, and dopamine, which impact neurons in the brain, spinal cord, and other cells in the airways. Additionally, they secrete hormones like CGRP, GRP, and somatostatin, affecting cells in various body parts, including the brain, lungs, and gut (Assas et al., 2014). PNECs are connected to the brain via the vagus nerve, a crucial link for normal respiration. Recent studies have identified two distinct groups of mouse vagus nerve afferents, P2ry1 and Npy2r, consisting of a few hundred neurons each, that have opposite effects on breathing. These neurons, which densely cover the lungs and connect to different areas in the brainstem, play contrasting roles. Npy2r neurons, primarily slow-conducting C fibers, contrast with the mainly fast-conducting A fibers of P2ry1 neurons, which interact with pulmonary neuroepithelial bodies. Optogenetic activation of P2ry1 neurons significantly inhibits respiration, causing animals to remain in an exhaled state, while stimulation of Npy2r neurons induces rapid and shallow breathing. Importantly, P2ry1 neuron activation does not impact heart rate or gastric pressure, underscoring their specific physiological functions. This highlights the complexity of the vagus nerve, which contains mixed sensory neurons with unique genetic markers, anatomical pathways, and physiological roles (Chang et al., 2015).

Communication between the CNS and the lung has also been found to be mediated through exosomes, a type of extracellular vesicles with size in the range of 30-200 nm with a lipid bilayer membrane. Exosomes are secreted by a diverse range of cell types, such as neuronal cells, neuroendocrine cells, and cancer cells (Thakur et al., 2020b,^2021^, 2022a,2022c; Iyaswamy et al., 2023).They are also found in various biofluids such as blood, cerebrospinal fluids, pleural fluids, and can cross biological barriers such as blood-brain barrier, facilitating the autologous and heterologous intracellular communication, and making them an important tool for theranostic research (Thakur et al., 2020a,c, 2022b; Xu et al., 2021; Linlin Liu et al., 2022). Exosomes facilitate communication between the CNS and peripheral nerve cells via cerebrospinal fluid. They also bridge the peripheral circulation and the CNS through the blood-brain barrier. This suggests their pivotal role in connecting the brain and lung axis. Exosomes increase in the circulation after traumatic brain injury, and their contents have been linked to acute lung injury. Furthermore, exosomes have been linked to the spread of lung cancer in the CNS. Although much of the research on exosomes has been conducted *in vitro*, they are thought to significantly facilitate communication between the brain and lungs. However, direct evidence supporting this role remains limited (Bátiz et al., 2015; Couch et al., 2017; Gan et al., 2020; Kerr et al., 2020).

## Technological advancements that propelled PNECs studies

**FIGURE 2:**
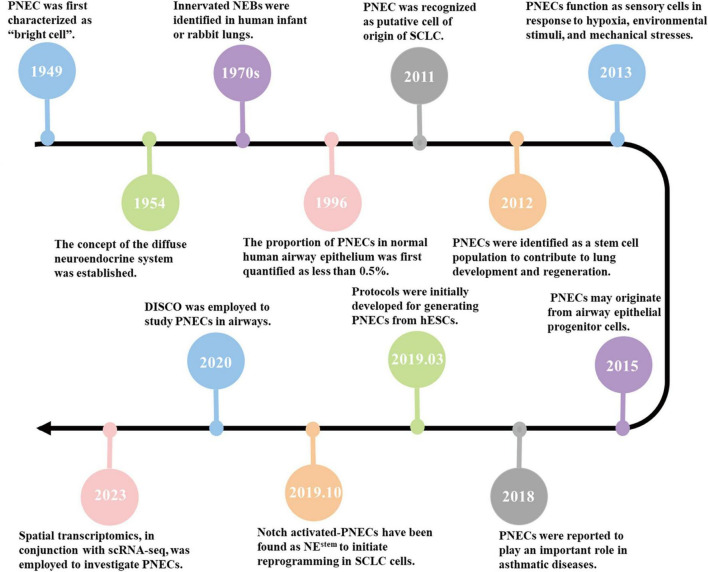
Timeline of PNECs research. 1949: PNECs were first characterized as ‘bright cells’ (Frohlich, 1949). 1954: The concept of the diffuse neuroendocrine system was established (Feyrter, 1954). 1970s: Innervated NEBs were identified in human infant or rabbit lungs (Lauweryns and Peuskens, 1972; Lauweryns et al., 1973). 1996: The proportion of PNECs in normal human airway epithelium was first quantified as less than 0.5% (Boers et al., 1996). 2011: PNEC was recognized as putative cell of origin of SCLC (Sutherland et al., 2011). 2012: PNECs were identified as a stem cell population to contribute to lung development and regeneration (Song et al., 2012). 2013: PNECs function as sensory cells in response to hypoxia, environmental stimuli, and mechanical stresses (Cutz et al., 2013). 2015: PNECs may originate from airway epithelial progenitor cells (Kuo and Krasnow, 2015). 2018: PNECs were reported to play an important role in asthmatic diseases (Sui et al., 2018). 2019.03: Protocols were initially developed for generating PNECs from hESCs (Chen et al., 2019). 2019.10: Notch activated-PNECs have been found as NE^stem^ to initiate reprogramming in SCLC cells (Ouadah et al., 2019). 2020: DISCO was employed to study PNECs in airways (Liu et al., 2020). 2023: Spatial transcriptomics, in conjunction with scRNA-seq, was employed to investigate PNECs (Sountoulidis et al., 2023).

Since the initial identification and description of PNECs, numerous technological advancements have emerged. These include stem cell technology (Chen et al., 2019), single-Cell RNA sequencing (Chen et al., 2019; Travaglini et al., 2020; Cai et al., 2021; Kuo et al., 2022), and spatial transcriptomics (Wang et al., 2023), immunolabelling enabled-3 Dimension Imaging of Solvent-cleared Organs (iDISCO) (Line Verckist et al., 2020; Liu et al., 2020; Sountoulidis et al., 2023), and four dimensional imaging by two-photon microscopy (Noguchi et al., 2015). Figure 2 provides a timeline highlighting key milestones in PNEC research.

### Stem cell technology

Studying human PNECs is challenging due to their rarity. Stem cell-derived PNECs offer valuable insights into their development and differentiation (Lian et al., 2022). Our research using human embryonic stem cell (hESC)-based models revealed that inhibiting NOTCH signaling and reducing RB protein levels can lead to the development of PNECs, which may give rise to small cell lung cancer (SCLC) (Chen et al., 2019). Additionally, introducing cMYC transgenes accelerated tumor growth and metastasis (Chen et al., 2023). Similar studies by Hor et al. using iPSC-derived PNECs (iPNECs) showed improved differentiation efficiency and alignment with human fetal PNECs when inhibiting Notch signaling (Hor et al., 2020a,b). These findings highlight the potential of stem cell-derived models to enhance our understanding of PNEC biology and their role in diseases such as SCLC.

### Single-Cell RNA Sequencing and spatial transcriptomics

Single-cell RNA sequencing (scRNA-seq) has revolutionized PNECs studies by enabling high-resolution gene expression analysis. Combining scRNA-seq with spatial transcriptomics provides comprehensive insights into cellular differentiation and maturation (Zhang et al., 2022). Studies by Kuo and Alexandros have identified unique gene expression programs associated with PNEC differentiation, advancing our understanding of these cells’ development and function (Kuo et al., 2022; Sountoulidis et al., 2023).

### Three dimensions imaging of un-sectioned lung by iDISCO technique

iDISCO enables comprehensive immunolabeling and 3D imaging of large tissue samples (Renier et al., 2014). Studies have demonstrated its effectiveness in visualizing CGRP-positive neuroendocrine cells and conducting multiple immunostainings in mouse lungs (Line Verckist et al., 2020; Liu et al., 2020; Brouns et al., 2023). This technique facilitates detailed studies of PNECs and NEBs, providing valuable insights into their structure and function.

### Four-dimensional imaging by two-photon microscopy

This method tracks PNEC migration using live imaging, integrating air-liquid culture with two-photon microscopy, allowing continuous observation of PNECs over time.

## Research perspectives on the role of PNECs in brain diseases

**FIGURE 3:**
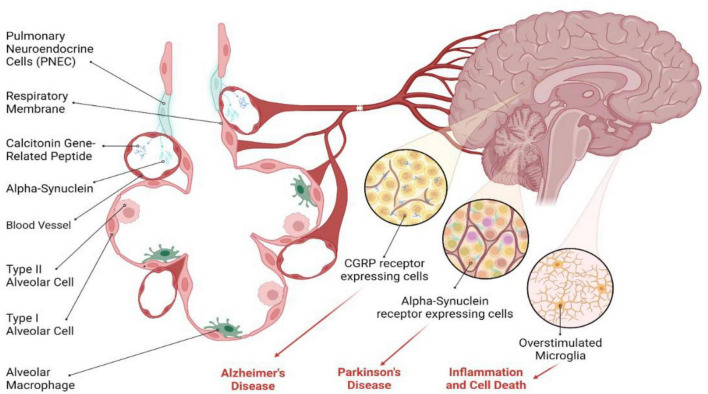
Potential role of PNECs in brain diseases. PNECs have been implicated in neurodegenerative diseases, including Alzheimer’s disease (AD) and Parkinson’s disease (PD). While direct evidence is lacking, PNECs appear to modulate neural activity through the release of CGRP, a neuropeptide that serves as a potent mediator of various physiological processes, including inflammation and cholinergic neurotransmission. In the context of AD, CGRP enhances cholinergic activity and acts as an anti-inflammatory agent, which may be relevant to AD pathogenesis. In PD, PNECs’ ability to modulate neuronal sensitivity through CGRP may have downstream effects on α-Synuclein aggregation, contributing to PD pathology. Several studies indicate the role of CGRP in different neuropathological conditions in the brain, suggesting a potential role for PNEC-derived secretory molecules in neurodegenerative diseases. However, further research is needed to explore the exact role of PNECs in these conditions.

PNECs are emerging as significant players in the intricate relationship between the respiratory system and neurodegenerative diseases, such as Alzheimer’s disease (AD) and Parkinson’s disease (PD) (Figure 3). PNECs communicate airway status to the brain via sensory neurons and local release of neuroactive substances like CGRP and GABA (Reddington et al., 1995; An et al., 2021; Gimeno-Ferrer et al., 2022; Kuo et al., 2022). These cells also secrete serotonin and bombesin-like peptides, which can enter the bloodstream and influence brain function by acting on central nervous system receptors (Corrin and Nicholson, 2011). Chronic lung inflammation, exacerbated by PNEC activity, can lead to systemic inflammation, a known contributor to cognitive decline and mood disorders (Suman et al., 2022; Sun et al., 2022). This systemic inflammation, driven by cytokines and other signaling molecules, may form a link between respiratory health and neurological conditions, suggesting that PNECs play a broader role in overall neuroinflammatory pathways (Sui et al., 2018).

In the context of AD, CGRP released by PNECs could impact neural activity by modulating cholinergic functions and serving as an anti-inflammatory agent (Assas et al., 2014). The presence of CGRP in nerve fibers and immune cells indicates its potential influence on AD pathogenesis. For PD, characterized by α-Synuclein (α-Syn) aggregates in dopaminergic neurons (Stefanis, 2012), the role of PNECs remains speculative but intriguing (Choi et al., 2022). While a direct connection between PNECs and α-Syn aggregation is yet to be established, the ability of PNECs to alter neuronal sensitivity via CGRP might indirectly affect PD pathology (Noguchi et al., 2020). Moreover, CGRP’s potential to cross the blood-brain barrier and modulate microglial activation and neuroinflammatory responses in neurodegenerative diseases underscores the need for further investigation. The exploration of PNECs’ secretory molecules and their transport via exosomes opens new research avenues, potentially unveiling novel mechanisms and therapeutic targets for neurodegenerative diseases.

## Conclusion

PNECs are a rare but vital cell type in the lungs, performing various roles in both healthy and diseased states. They develop from embryonic lung epithelial progenitors and are involved in numerous lung functions and diseases. PNECs also act as sensory cells, communicating changes in airway conditions to the brain and playing roles in mucociliary clearance, airway tone, and the development of asthma and bronchial hyperresponsiveness. Additionally, they are involved in lung cancer and repair damaged airway epithelium. PNECs may serve as a therapeutic target for various lung diseases, including asthma and COPD. They communicate with the brain via neural connections and neurotransmitter release, possibly influencing conditions like AD and PD. The proliferation of PNECs is a challenge in lung diseases, and stem cell technology may aid in their study. Future strategies may target PNECs for therapeutic benefits in lung diseases and explore their role in airway-nerve communication. As sensory cells, PNECs alert the brain about airway changes and external stimuli. They regulate mucociliary clearance, airway tone, and are involved in asthma and bronchial hyperresponsiveness. PNECs are also linked to lung cancer and repairing damaged airway epithelium, making them potential targets for treating respiratory diseases.

PNECs are instrumental in facilitating communication between the lungs and brain. This occurs through direct neural links to the brainstem and spinal cord, and by releasing neurotransmitters and hormones. PNECs play a vital role in regulating essential autonomic functions such as breathing and heart rate. They release neurotransmitters like CGRP, which influences neural activity and immune responses. Furthermore, PNECs might be involved in neurodegenerative diseases like AD and PD, possibly through the impact of CGRP on cholinergic activity and inflammation.

The proliferation of PNECs is a significant issue in many lung diseases. To tackle this issue, stem cell technologies, such as human Embryonic Stem Cells and induced Pluripotent Stem Cells, are employed to generate a sufficient quantity of PNECs for research purposes. This approach is particularly useful given the rarity of human PNECs and the lack of established isolation techniques. These advanced technologies are crucial for exploring the pathophysiology of PNECs in various lung disorders.

In the future, developing strategies to target PNECs could offer new therapeutic options for lung diseases, including SCLC and asthma. Given the dual neuronal and endocrine nature of PNECs, it’s crucial to explore their potential role as a communication channel between the lungs and brain. This includes understanding their involvement in both anterograde and retrograde neurotransmission. Investigating how disturbances in PNECs might disrupt signal transmission to the brain in pathological conditions could provide significant insights into lung-brain interactions.

## Author contributions

AT: Conceptualization, Supervision, Writing−original draft, Writing−review and editing, Visualization. SM: Writing−review and editing. NZ: Resources, Writing−review and editing. KZ: Resources, Writing−review and editing. BT: Resources, Writing−review and editing. JL: Resources, Writing−review and editing. SW: Resources, Writing−review and editing. BC: Writing−review and editing. JS: Writing−review and editing. HC: Conceptualization, Funding acquisition, Supervision, Writing−review and editing.
